# The ascidian natural product eusynstyelamide B is a novel topoisomerase II poison that induces DNA damage and growth arrest in prostate and breast cancer cells

**DOI:** 10.18632/oncotarget.6267

**Published:** 2015-11-02

**Authors:** Michelle S. Liberio, Martin C. Sadowski, Rohan A. Davis, Anja Rockstroh, Raj Vasireddy, Melanie L. Lehman, Colleen C. Nelson

**Affiliations:** ^1^ Australian Prostate Cancer Research Centre – Queensland, Institute of Health and Biomedical Innovation, Queensland University of Technology, Princess Alexandra Hospital, Translational Research Institute, Brisbane, Queensland, Australia; ^2^ Eskitis Institute for Drug Discovery, Griffith University, Nathan, Queensland, Australia; ^3^ Vancouver Prostate Centre, University of British Columbia, Vancouver, British Columbia, Canada

**Keywords:** eusynstyelamide B, G2/M arrest, DNA damage, topoisomerase II poison, LNCaP

## Abstract

As part of an anti-cancer natural product drug discovery program, we recently identified eusynstyelamide B (EB), which displayed cytotoxicity against MDA-MB-231 breast cancer cells (IC_50_ = 5 μM) and induced apoptosis. Here, we investigated the mechanism of action of EB in cancer cell lines of the prostate (LNCaP) and breast (MDA-MB-231). EB inhibited cell growth (IC_50_ = 5 μM) and induced a G2 cell cycle arrest, as shown by a significant increase in the G2/M cell population in the absence of elevated levels of the mitotic marker phospho-histone H3. In contrast to MDA-MB-231 cells, EB did not induce cell death in LNCaP cells when treated for up to 10 days. Transcript profiling and Ingenuity Pathway Analysis suggested that EB activated DNA damage pathways in LNCaP cells. Consistent with this, CHK2 phosphorylation was increased, p21^CIP1/WAF1^ was up-regulated and CDC2 expression strongly reduced by EB. Importantly, EB caused DNA double-strand breaks, yet did not directly interact with DNA. Analysis of topoisomerase II-mediated decatenation discovered that EB is a novel topoisomerase II poison.

## INTRODUCTION

During an anti-cancer natural product drug discovery program [1, 2], we recently identified eusynstyelamide B (EB) from the Great Barrier Reef marine ascidian, *Didemnum candidum.* This complex and unique *bis*-indole alkaloid displayed cytotoxicity (IC_50_ = 5 μM) and induced apoptosis in MDA-MB-231 breast cancer cells [3]. Marine organisms such as sponges and ascidians have been a prolific source of cytotoxic compounds several of which have been shown to target topoisomerase enzymes. Marine natural products belonging to the makaluvamine, pyridoacridine and xestoquinone structure classes have all been shown to interact and perturb topoisomerases [4]. The discovery of novel cytotoxic compounds is very important for the development of anti-cancer treatments [5]. New cytotoxic drugs have been recently approved (eribulin, trabectedin, ixabepilone) and many are being tested in the clinic against chemoresistant cancers and in drug combination therapies [5–8].

Topoisomerase poisons are among the most widely prescribed anti-cancer drugs in clinical use. These cytotoxic drugs (e.g. etoposide, doxorubicin, and mitoxantrone) are frontline therapies for a variety of cancers [9, 10]. Topoisomerases are essential nuclear enzymes that play a major role in DNA replication, transcription, recombination, chromosome condensation and segregation [9, 11–13]. There are two major topoisomerase families. Type I topoisomerases make transient cuts in the DNA, regulating over- and under-winding within the double helix which reduces the stress accumulated ahead of replication forks and transcription complexes. Type II topoisomerases make transient double-strand breaks in DNA and modulates under- and over-winding, knotting, and tangling. Topoisomerase II can be found in two forms, topoisomerase IIα and IIβ [9, 11–13]. These isoforms are differentially expressed in cells and have separate nuclear functions. Topoisomerase IIα is regulated through cell cycle and its maximal level peaks at the G2/M boundary. Moreover, this isoform is found in rapidly proliferating tissues and can be found in replication forks and associated with chromosomes during mitosis [9, 11–13]. In contrast, the β isoform is present in most cell types independent of their proliferation status and it appears to be involved in the transcription of hormonally and developmentally regulated genes [14, 15].

Topoisomerase II-inhibiting drugs can affect different stages of the catalytic cycle and are categorized into two groups: catalytic inhibitors and poisons. Catalytic inhibitors prevent the formation of the cleavage complex through inhibition of TOPO II binding caused by its intercalation into DNA [9, 11–13, 16]. The bisdioxopiperazines, ICRF- 187 and ICRF-193 and the quinoline aminopurine are examples of catalytic inhibitors that stabilize the closed clamp intermediate, which is formed by the enzyme around the DNA, and blocks ATP hydrolysis [17, 18]. In contrast, TOPO II poisons stabilize the cleavage complex [9, 11– 13, 19], and can be categorized as interfacial or covalent [20, 21]. The interfacial poisons etoposide, doxorubicin, mitoxantrone, and bioflavonoids such as genistein bind non-covalently to the cleavage complex, intercalate into the DNA at the cleaved scissle bond and prevent religation. Covalent poisons have protein reactive groups, such as quinones, isothiocyanates, and maleimides that form adducts with the enzyme. The stabilization of the DNA cleavage complex leads to the formations of permanent double strand breaks when, for example, replication forks and transcription complexes try to transverse the cleavage. This can cause genome instability and chromosome translocations, which is associated with the development of some specific forms of leukemia [10, 22]. Currently, no drugs specific to topoisomerase IIα or β are available for clinical use. Results suggest that cardiotoxicity resulting from the use of the topoisomerase II-targeted drugs doxorubicin is due to its interactions with the β isoform [23]. There is also evidence that this isoform is responsible for initiating some of the secondary malignancies associated with topoisomerase-targeted drugs [24]. Compounds such as, NK314, tricitrinol B and Dp44mT favor TOPO IIα and aim for producing less off-targeted effects [25–28]. At the moment, four TOPO II-targeted drugs are in clinical development: F14512, versaroxin, C-1311 and XK469 [10].

Here, we report mechanism of action studies on eusynstyelamide B (EB), providing a basis for further development of this agent (or optimized analogs) as a potential human breast and prostate cancer therapeutic. Our data indicated that EB inhibited the proliferation of LNCaP and MDA-MB-231 cells *in vitro* by inducing a G2 arrest. Importantly, EB was found to be a non-intercalating topoisomerase II poison that activates DNA damage response pathways.

## RESULTS

### EB arrested growth of LNCaP cells

**Figure 1:**
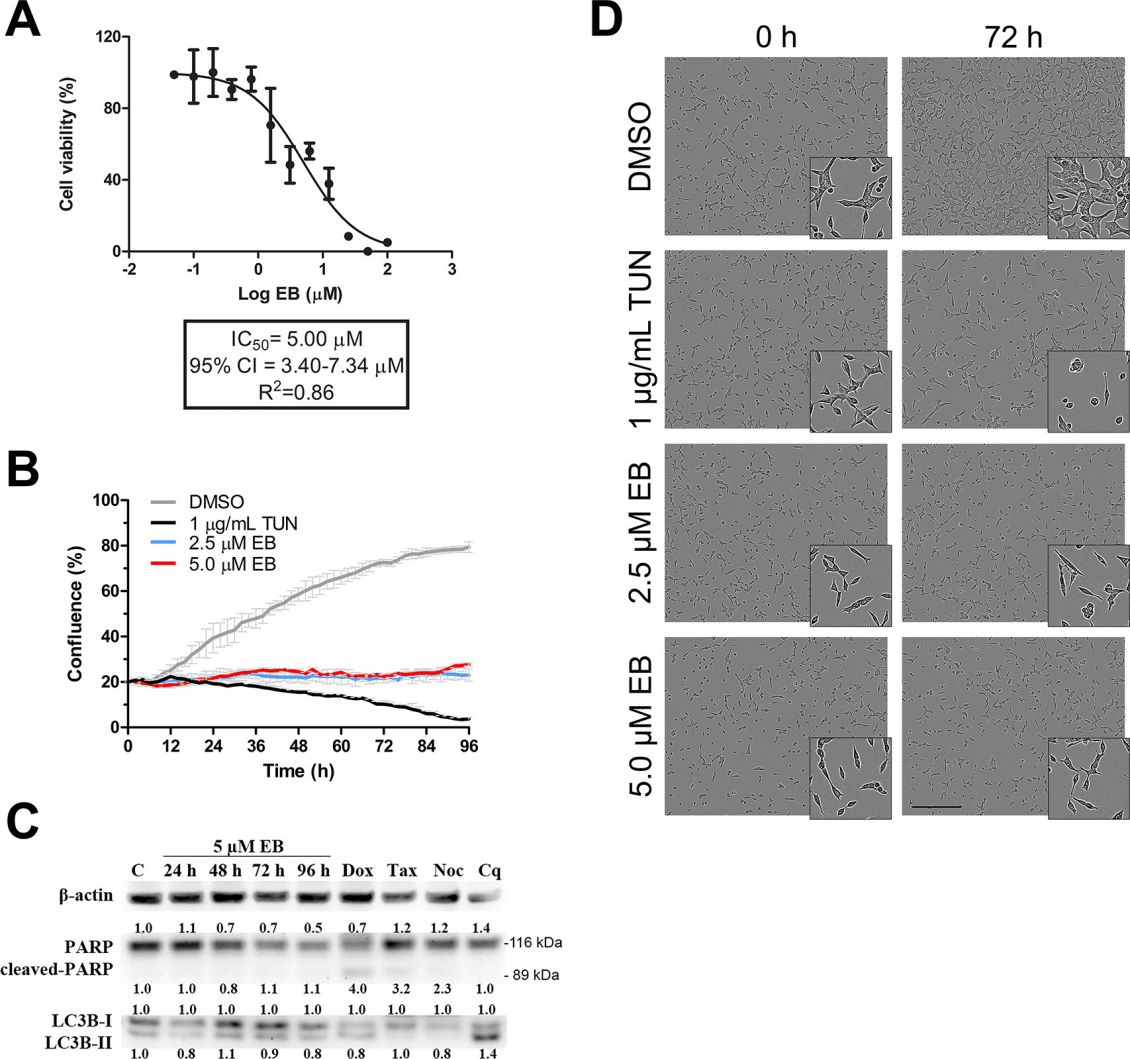
EB arrested growth of LNCaP cells **(A)** LNCaP cells were treated with the indicated concentrations of EB, and growth was monitored with a real-time cell analyzer (xCELLigence) for 72 h in three independent experiments. The IC_50_ was calculated by non-linear regression analysis of the dose response curves (*n* = 3, mean ±SD). **(B)** LNCaP cells were treated with 2.5 μM EB, 5.0 μM EB, 1.0 μg/mL tunicamycin (TUN, positive control), or vehicle control (DMSO). Cell growth as a function of increasing confluence was measured by real-time phase contrast imaging every two hours for 96 h on a live cell IncuCyte FLR system (*n* = 3, mean ±SD). **(C)** LNCaP cells were treated with 5.0 μM EB for the indicated times after which protein lysates were prepared and analyzed by Western blot analysis for the levels of PARP (116 kDa), cleaved PARP (89 kDa), LC3B-I (16 kDa), LC3B-II (14 kDa), and β-actin as a loading control. Control (C) cells were treated with the drug vehicle DMSO (0.1%) for 96 h. Other controls used were doxorubicin (Dox, 1 μM for 48 h), taxol (Tax, 2 nM for 24 h), and nocodazole (Noc, 83 nM for 24 h) as positive controls for PARP cleavage and chloroquine (Cq, 25 μM for 48 h) as a positive control for autophagy. Protein levels were quantified, normalized against the loading controls, and the results were expressed in relation to DMSO control (C). **(D)** Representative images of the analysis in B after 0 h and 72 h of treatment.

We recently demonstrated during a screening campaign of an ascidian-derived extract library that EB inhibited growth (IC_50_ 5.0 μM) and caused cell death through apoptosis in MDA-MB-231 breast cancer cells [3]. As shown in Figure 1A, analysis of growth with a real-time cell analyzer (xCELLigence) revealed that EB exhibited a similar inhibitory potency in the prostate cancer cell line LNCaP (IC_50_ 5.0 μM). Real time analysis of cell confluence by live cell imaging (IncuCyte FLR) demonstrated that 2.5 μM and 5.0 μM EB efficiently blocked growth of LNCaP cells up to 96 h (Figure 1B). Yet, no typical morphological signs of cell death (cell shrinkage and membrane blebbing) were observed after 96 h (Figure 1C) or 10 days of treatment (Figure S1), suggesting that EB is cytostatic in LNCaP cells (36 h doubling time). Indeed, Western blot analysis of LC3B-II, a marker of autophagy, and cleaved PARP, a marker of late apoptosis, as well as Annexin V staining, a marker of early apoptosis (data not shown), confirmed that EB did not induce autophagy or apoptosis in LNCaP cells (Figure 1D). Notably, growth of the highly proliferative primary human neonatal foreskin fibroblast cell line NFF (IC_50_ 1.3 μM, 24 h doubling time) and non-malignant prostate cell line RWPE-1 (IC_50_ 0.92 μM, 22 h doubling time) was also inhibited by EB (Figure S2), suggesting that EB displayed higher potency in fast proliferating cell lines.

### EB induced a G2 cell cycle arrest

**Figure 2:**
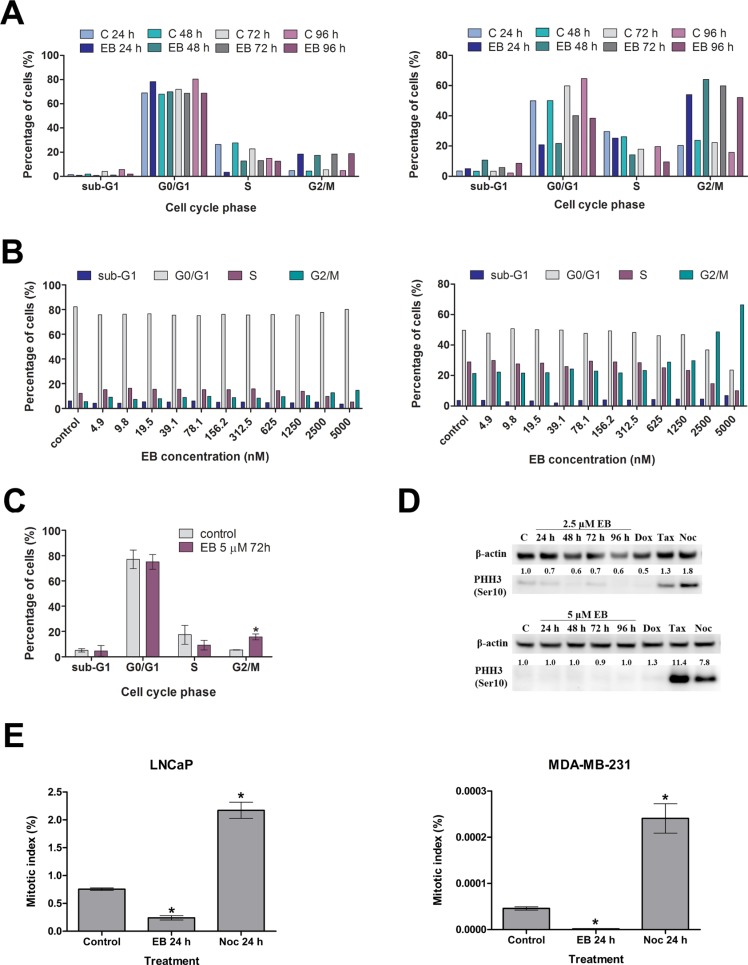
EB induced a G2 cell cycle arrest **(A)** Cell cycle distribution of LNCaP (left panel) and MDA-MB-231 cells (right panel) treated for the indicated times with 5 μM EB or 0.1% DMSO (control). DNA content was analyzed by flow cytometry, and the number of cells in the indicated cell cycle phases was quantitated. **(B)** LNCaP (left panel) and MDA-MB-231 cells (right panel) were treated for 72 h with the indicated concentrations of EB and analyzed as in A. **(C)** LNCaP cells were treated for 72 h with 5 μM EB or 0.1% DMSO (control) and analyzed as in A (*n* = 3, mean ± SD, *p < 0.05). **(D)** MDA-MB-231 (top panel) and LNCaP cells (bottom panel) were treated with 2.5 μM and 5 μM EB, respectively, and extracted at the indicated time points for Western blot analysis with anti-phospho-histone H3 antibody (PHH3). β-actin levels were determined as loading control. As a control (C), cells were treated with the drug vehicle DMSO (0.1%) for 96 h. Other controls used were the DNA damage inducer doxorubicin (Dox, 1 μM for 48 h), and the anti-mitotic drugs taxol (Tax, 2 nM for 24 h) and nocodazole (Noc, 83 nM for 24 h). Protein levels were quantified, normalized against the loading controls, and the results were expressed in relation to the DMSO control (C). **(E)** Quantification of the mitotic index by HCS after phospho-histone H3 labelling. LNCaP and MDA-MB-231 cells were treated with 5 μM EB for 24 h and probed with anti-phospho-histone H3 antibody. Control cells were treated for 24 h with 0.1% DMSO or 83 nM of nocodazole. Quantification of PHH3 staining and calculation of the mitotic indices were carried out on the HCS instrument Operetta (PerkinElmer). Asterisks indicate results with *p* < 0.05 in a One-way ANOVA analysis.

Previous work by our group described a significant G2/M arrest of MDA-MB-231 breast cancer cells after treatment with 5.0 μM EB for 72 h [3]. A time course study of MDA-MB-231 and LNCaP cells revealed that EB induced a G2/M arrest in both cell lines as early as 24 h after treatment had commenced (Figure 2A). Concomitant with the increase of the G2/M cell population, EB largely reduced the G0/G1 cell population of MDA-MB-231 cells with a modest decrease of the number of cells in S phase, while EB mainly affected the S phase cell population in LNCaP cells. Furthermore, the G2/M arrest of MDA-MB-23 cells was most pronounced after 48 h, after which the number of cells in G2/M visibly declined and the G0/G1 cell population increased, suggesting that the inhibitory effect of EB was in part temporary in the breast cancer cell line (Figure 2A). In contrast, the EB-induced G2/M arrest remained unchanged in LNCaP cells over the treatment period of 96 h (Figure 2A) and increased after 10 days of treatment (Figure S1). EB-treated MDA-MB-231 cells consistently displayed higher levels of dead cells with hypodiploid DNA content (sub-G1) compared to control when treated for 48 h or longer, while no such trend was visible in LNCaP cells (Figure 2A) even after 10 days of treatment (Figure S1). A dose titration experiment (4.9 nM to 5 μM EB) for 72 h showed that concentrations of 0.625 μM EB and higher induced a visible increase of the G2/M cell population of MDA-MB-231 cells, while 5 μM EB were required to visibly arrest LNCaP cells in G2/M (Figure 2B). Similar to the results above, there was a modest concentration-dependent increase in the number of dead cells (sub-G1) in the breast cancer cell line but not in LNCaP cells (Figure 2B). Treatment of LNCaP cells with 5 μM EB for 72 h confirmed that EB significantly increased the number of cells in G2/M (*p* < 0.05) (Figure 2C).

With the purpose of determining if EB arrested LNCaP and MDA-MB-231 cells in G2 or in M phase of the cell cycle, two different experimental approaches were pursued. First, cell lysates of EB-treated cells were probed for the expression of the mitotic marker phospho-histone H3 (PHH3) by Western blotting. Elevated levels of PHH3 indicate an increase in the amount of cells in mitosis [29]. This was observed with MDA-MB-231 and LNCaP cells when treated with taxol or nocodazole, which both arrest cells in mitosis (Figure 2D) [30, 31]. In contrast, EB treatment decreased the phosphorylation of histone H3 in MDA-MB-231 cells (Figure 2D), while PHH3 levels remained unchanged in LNCaP cells (Figure 2D). The absence of increased PHH3 levels in both cell lines indicated that EB did not cause a mitotic arrest.

Next, the mitotic index of EB-treated MDA-MB-231 and LNCaP cells was calculated by high content screening (HCS) based on PHH3 staining. EB treatment decreased the mitotic index in MDA-MB-231 cells by 30-fold and in LNCaP cells by 3-fold (Figure 2E). Taken together, the increased G2/M cell population, absence of elevated PHH3 levels and reduced mitotic index after EB treatment together indicated that EB arrested LNCaP and MDA-MB-231 in G2 of the cell cycle.

### EB treatment activates the DNA damage response pathway

**Figure 3:**
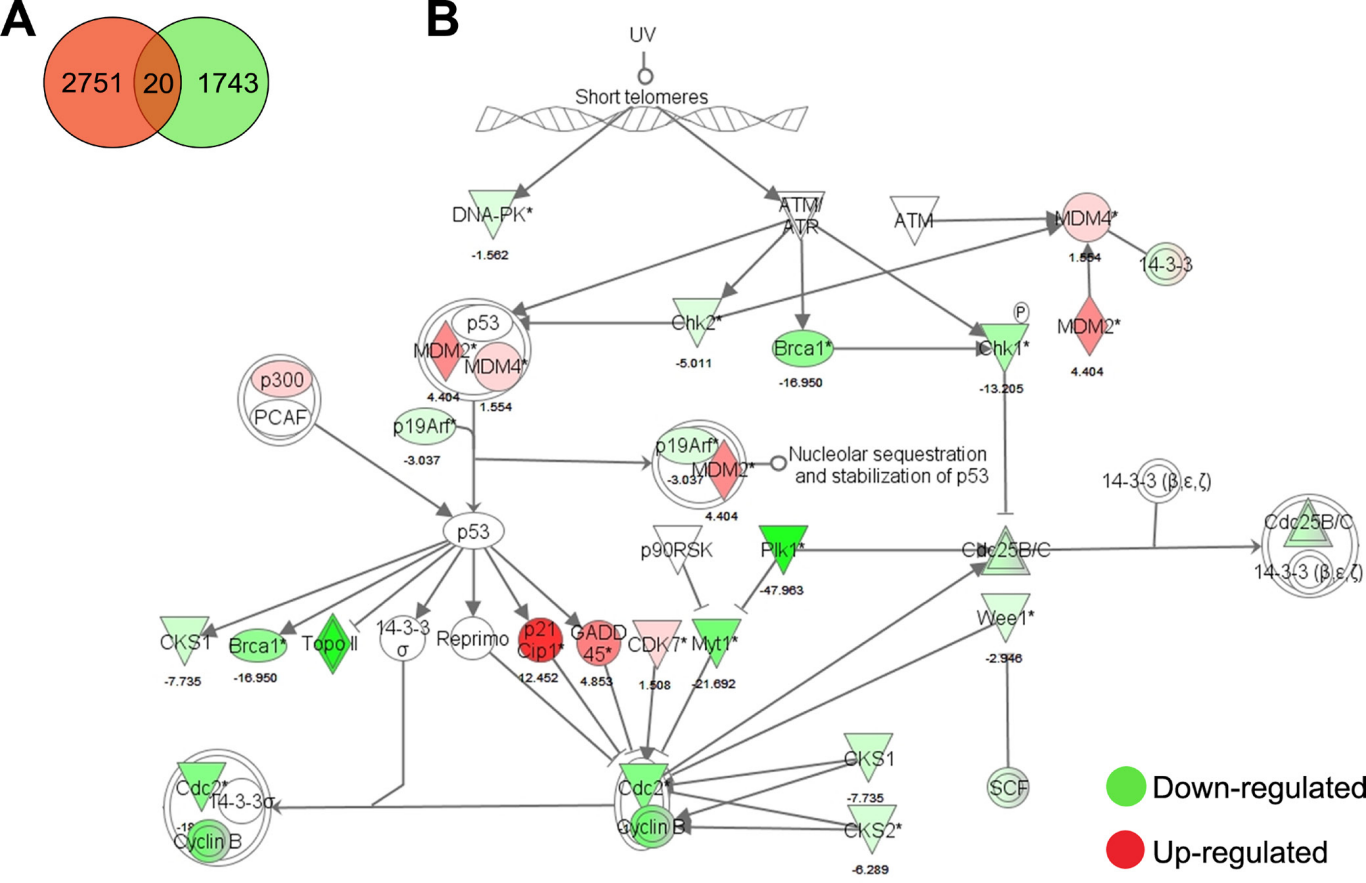
Microarray analysis of EB-treated LNCaP cells **(A)** Venn diagram of differentially expressed genes of LNCaP cells treated with 5.0 μM EB for 24 h. **(B)** Ingenuity pathway analysis of G2/M DNA damage checkpoint regulation. EB caused up-regulation of 2751 genes and down-regulation of 1743 genes. The canonical pathway of G2/M DNA damage checkpoint regulation was overlaid with the microarray analysis-derived gene expression changes of LNCaP cells treated with 5.0 μM EB for 24 h. Genes in red were up-regulated and genes in green were down-regulated by the indicated values (fold change) relative to vehicle control (DMSO).

To guide the characterization of the molecular mechanism underlying the EB-induced growth arrest of LNCaP cells, differential gene expression was studied by DNA microarray with a custom 180 k Agilent oligo microarray (VPCv3, ID032034, GPL16604). This prostate cancer focused array contains probes mapping to human protein-coding as well as non-coding loci; with probes targeting exons, 3′UTRs, 5′UTRs, intronic and intergenic regions [124]. With cut-offs of *p* ≤ 0.05 and fold change ≥ 1.5, EB caused up-regulation of 2751 genes and down-regulation of 1743 genes (Figure 3). The 20 most differentially regulated genes after EB treatment of LNCaP cells relative to DMSO control are shown in Table S1.

Pathway analysis of differentially regulated genes with Ingenuity IPA software indicated that EB strongly changed the expression of 615 genes involved in cell cycle regulation and of 504 genes involved in DNA replication, recombination and repair. Many genes related to the role of *BRCA1* in DNA damage response, such as *CHEK2* and *PLK1*, were substantially down-regulated (17- and 48-fold, respectively). Pathways related to DNA repair, including homologous and non-homologous recombination, showed down-regulation of most of its genes. Furthermore, ATM signaling, which comprises part of the DNA damage response, was another pathway highly de-regulated by EB (Figure 3). EB treatment affected 28 out of 47 genes related to G2/M check point regulation and 44 genes out of 99 involved in p53 signaling, respectively. For example, EB treatment increased *CDKN1A* expression by 12-fold and *MDM2* by 4-fold, and decreased BRCA1 expression by 17-fold and *CHEK1* by 13-fold.

### Validation of microarray results by qRT-PCR and Western blotting analyses

**Figure 4:**
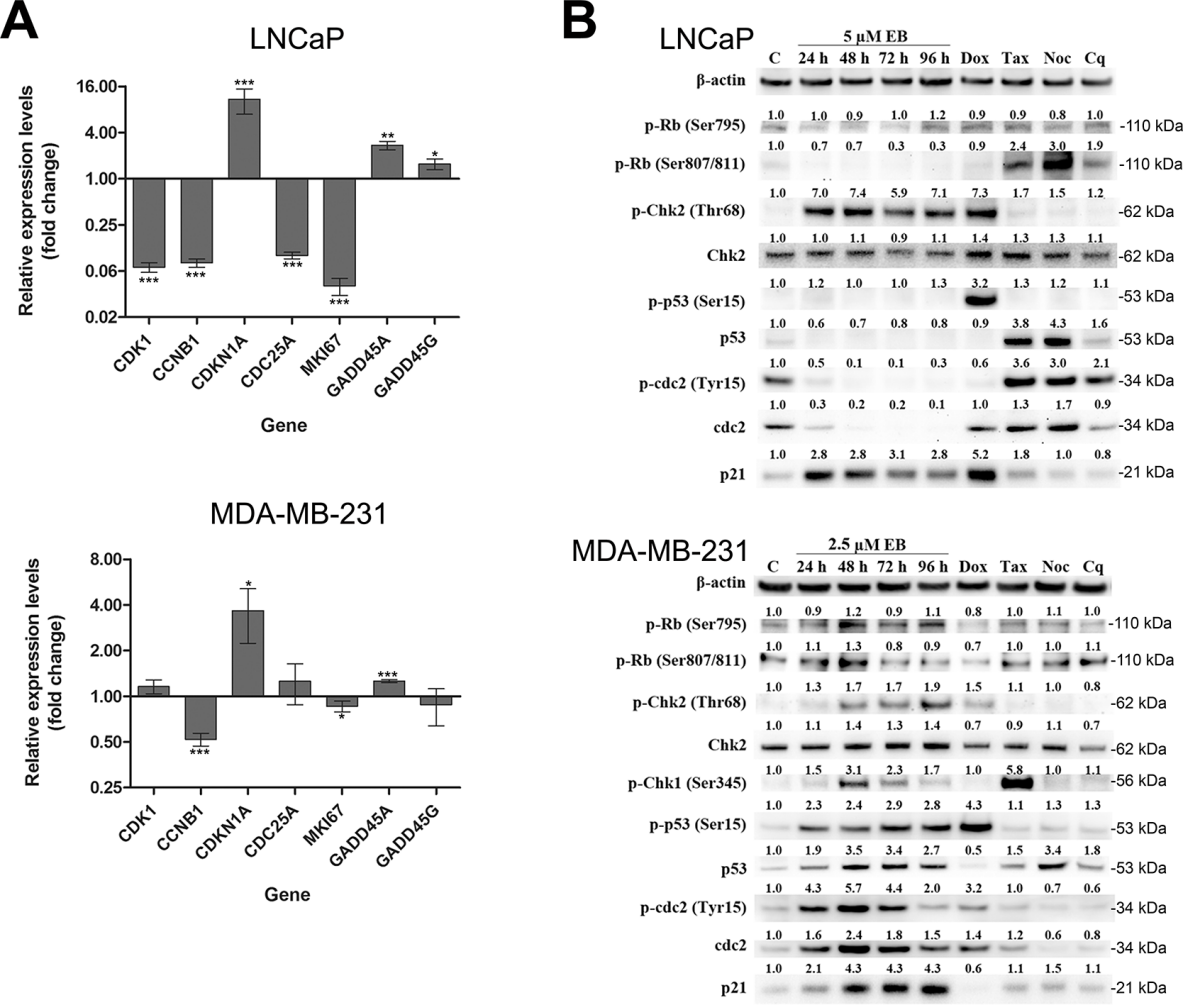
EB affected critical regulators of the G2/M DNA damage checkpoint and cell cycle **(A)** LNCaP and MDA-MB-231 cells were treated for 24 h with 5 μM EB prior to RNA extraction and analysis of gene expression by qRT-PCR of the indicated genes. As a control, cells were treated with 0.1% DMSO for 24 h. Expression levels are shown as fold change relative to control (*n* = 3, mean ± SD, *p < 0.05, **p < 0.001, ***p < 0.0001). **(B)** LNCaP and MDA-MB-231 cells were treated with 5 μM and 2.5 μM EB, respectively, and extracted at the indicated time points for Western blot analysis with antibodies directed against the indicated proteins. β-ACTIN levels were determined as loading control. As a control (C), cells were treated with the drug vehicle DMSO (0.1%) for 96 h. Other controls used were the DNA damage inducer doxorubicin (Dox, 1 μM for 48 h), the anti-mitotic drugs taxol (Tax, 2 nM for 24 h) and nocodazole (Noc, 83 nM for 24 h), and the autophagy inhibitor chloroquine (Cq, 25 μM for 48 h). Protein levels were quantified, normalized against the loading controls, and the results were expressed relative to the DMSO control (C).

Gene expression profiling by microarray analysis showed a strong differential regulation of genes involved in the control of cell growth, cell cycle and DNA damage response pathways. As shown by microarray analysis, the cell cycle regulation genes *CDK1*, *CCNB1* and *CDC25A* were transcriptionally down-regulated by 14-, 12-, and 9-fold, respectively, while the CDK inhibitor *CDKN1A* which encodes the p21^CIP1/WAF1^ protein, was up-regulated 12-fold. Similar levels of differential expression were observed by qRT-PCR (Figure 4A). Furthermore, expression of *MKI67*, which encodes the nuclear proliferation marker KI67, was suppressed 15-fold and 25-fold as shown by the microarray and qRT-PCR analyses, respectively (Figure 4A). Consistent with the results of the microarray experiment, qRT-PCR demonstrated that the growth-arrest and DNA-damage-inducible stress response genes *GADD45A* and *GADD45G* were up-regulated by 3- and 2-fold, respectively (Figure 4A). In summary, qRT-PCR analysis of seven critical cell cycle regulatory genes confirmed the observation of the microarray study that EB-induced their differential expression in LNCaP cells. In MDA-MB-231 cells, EB treatment increased the expression of *CDKN1A* by 4-fold, while it decreased the expression of *CCNB1* by 1.9- fold (Figure 4A). In addition, the transcription levels of *CDK1*, *CDC25A*, *MKI67*, *GADD45A* and *GADD45G* did not change substantially (fold change < 1.5) after EB treatment, suggesting cell line-specific difference in the regulation of these genes.

In order to validate the gene profiling result and to further study the molecular basis of the EB induced G2 cell cycle arrest, the expression of proteins involved in DNA damage response and G2/M check point regulation was investigated in both cell lines by Western blotting analysis. As controls, cells were treated with the DNA intercalator doxorubicin, which induces DNA damage by stalling topoisomerase II, the mitotic inhibitors taxol and nocodazole that target tubulin polymerization, and the autophagy inhibitor chloroquine [31–34]. The retinoblastoma tumor suppressor protein, RB, regulates cell proliferation by controlling G1-S phase progression of the cell cycle through its inactivation by phosphorylation [35, 36]. DNA damage can lead to RB dephosphorylation, which causes a cell cycle arrest in G1 [37, 38]. In MDA- MB-231 breast cancer cells, EB treatment showed only moderate alterations in RB phosphorylation (Ser795, Ser807 and Ser811), indicating that G1-S phase progression was not affected by EB treatment (Figure 4B). On the other hand, the amount of phosphorylated RB at Ser807/811 reduced over time after treatment of LNCaP cells, while Ser795 phosphorylation remained unchanged (Figure 4B). It is unclear why these three CDK4/CYCLIN D target sites were differentially regulated in LNCaP cells. Nevertheless, loss in RB phosphorylation leads to RB activation and inhibition of S phase progression as indicated by the reduced number of cells in S phase (Figure 2). The mRNA levels of *TP53*, which encodes the p53 protein, did not change after EB treatment in LNCaP cells (data not shown). Protein p53 is activated by phosphorylation in the presence of cellular stress, and regulates the expression and activation of molecules associated with cell cycle arrest, apoptosis, DNA repair, senescence, and metabolism. Increased phosphorylation of p53 was not detected up to 96 h after treatment of LNCaP cells with EB (Figure 4B). Instead, EB treatment temporarily reduced the expression of p53. In stark contrast to LNCaP cells, p53 phosphorylation and total p53 expression were substantially up-regulated in a time dependent manner in MDA-MB-231 cells (Figure 4B). In the absence of cellular stress, p53 is expressed at low levels but upon stress stimuli like DNA damage is stabilized and activated by a series of post-translational modifications, such as phosphorylation by the kinases DNA-PK and ATM/ATR [39]. Like p53, CHK2 is also activated through phosphorylation by DNA-PK and ATM/ATR following DNA damage [40]. EB treatment of both cell lines led to increased CHK2 phosphorylation. While CHK2 phosphorylation increased by approximately two-fold in a time-dependent manner in MDA-MB-231 cells, it was strongly up-regulated (seven-fold) as early as 24 h post EB treatment in LNCaP cells (Figure 4B). Interestingly, the microarray results showed that *CHEK2* (CHK2) gene expression was down-regulated by 5-fold in EB-treated LNCaP cells; however, the expression of total CHK2 protein was not affected by EB treatment, as shown by Western blotting (Figure 4B). Activation of CHK1 kinase inactivates the phosphatase CDC25, which removes the inhibitory Tyr15 phosphorylation of CDC2 (CDK1) [41]. Increased phosphorylation of CHK1 was observed in breast cancer cells with a peak at 48 h post treatment (Figure 4B), whereas it was undetectable in LNCaP cells (data not shown). The CDC2/CYCLIN B complex is critical for the transition from G2 into mitosis. Entry of eukaryotic cells into mitosis is regulated by activation of CDC2 kinase through de-phosphorylation of CDC2 at Thr14 and Tyr15 [42–44]. EB treatment induced an accumulation of total CDC2 protein and inhibitory phosphorylation at Tyr15 in MDA-MB-231 cells (Figure 4B). When corrected for the up-regulation in total protein levels of CDC2, Tyr15 phosphorylation was increased by approximately 2-fold. The opposite was observed in EB-treated LNCaP cells (Figure 4B) where total CDC2 protein was markedly reduced at every time point. This is in agreement with the microarray and qRT-PCR results which showed a 14- and 17-fold reduction in CDC2 gene expression after EB treatment of LNCaP cells for 24 h. While the inhibitory Tyr15 phosphorylation of CDC2 was slightly increased after 24 h of EB treatment when corrected for the decline in total CDC2 protein levels, it was barely detectable at later time points, which was probably due to the strong loss of CDC2 protein. Consistent with the transcriptional changes of *CDKN1A* (p21^CIP1/WAF1^) (Figure 4A), expression of the kinase inhibitor was strongly induced in both cell lines after EB treatment (Figure 4B). The cyclin-dependent kinase inhibitor 1 (p21^CIP1/WAF1^) works as a cell cycle regulator of G1 and S phase as well as an important mediator of cell cycle arrest at G2/M phase in response to DNA damage [45]. The expression of p21^CIP1/WAF1^ is up-regulated in the presence of low levels of DNA damage; however, at high levels of DNA damage, p21^CIP1/WAF1^ is proteolytically removed followed by induction of apoptosis [45]. Taken together, qRT-PCR and Western blot analysis corroborated above findings of the cell cycle and microarray analyses. Importantly, they demonstrated that critical regulators of the DNA damage pathways (GADD45, p53, CHK1, and CHK2) were activated.

### EB caused DNA double strand breaks

**Figure 5:**
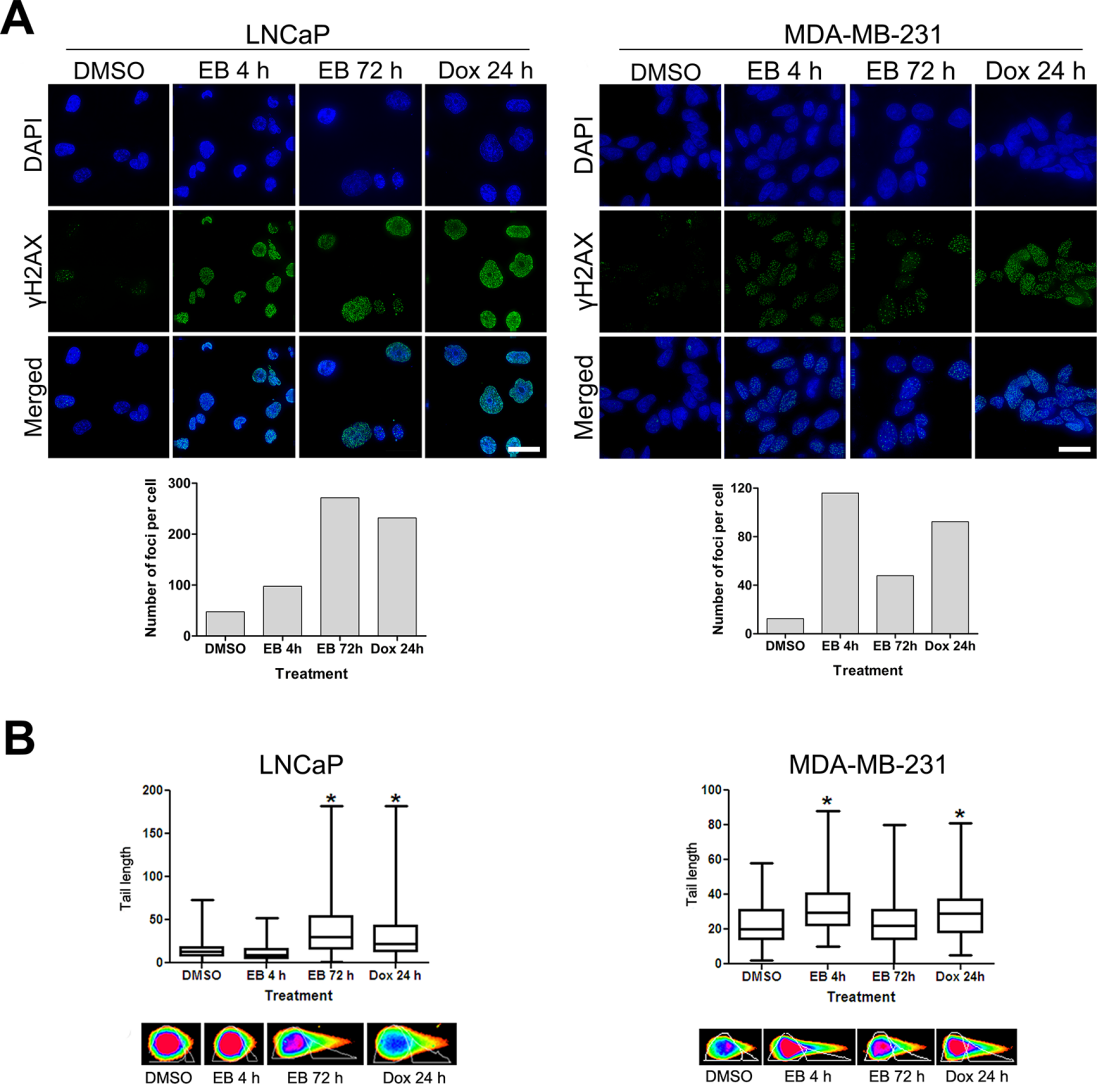
EB caused DNA double strand breaks **(A)** EB induced γH2AX foci formation. LNCaP and MDA-MB231cells were treated for 4 h and 72 h with 5 μM and 2.5 μM and EB, respectively. As controls, cells were treated with 0.1% DMSO for 72 h (control) or the DNA damage inducing agent doxorubicin (Dox, 5 μM) for 24 h. Fixed cells were reacted with antibodies directed against γH2AX (green), DNA was counterstained with DAPI (blue), and cells were visualized by immunofluorescence microscopy with a DeltaVision microscope (60 × objective). The number of foci per cell was quantified with Metamorph software (*n* < 100 cells, scale bar = 30 μm). **(B)** LNCaP and MDA-MB-231 cells were treated for 4 h and 72 h with 5 μM and EB, and DNA damage was analyzed by neutral single cell COMET assay. As controls, cells were treated with 0.1% DMSO for 72 h (control) or 5 μM doxorubicin (Dox) for 48 h. The amount of DSBs was quantified by measuring the length of the comet tails with CometScore software (*n* = 100 cells, *p < 0.05). Representative images of the comet tails are shown (bottom panels).

The above studies demonstrated that MDA-MB-231 and LNCaP cells reacted to EB treatment by differential regulation of genes and proteins involved in DNA damage pathways, suggesting that EB might induce DNA damage. In order to investigate whether EB causes DNA double strand breaks (DSBs), EB-treated LNCaP and MDA-MB-231 cells were analyzed by quantitative γH2AX foci and neutral COMET assays. DSBs induce phosphorylation of histone H2AX (γH2AX), generating foci in the nucleus that can be detected by specific antibodies [46]. In a time-dependent manner, EB substantially increased the number of γH2AX foci in LNCaP cells to levels seen after treatment with doxorubicin (Figure 5A). A strong increase in the number of γH2AX foci was also observed in MDA-MB-231 cells when treated for 4 h with EB. Yet, longer treatment periods (72 h) generated visibly less DSBs, suggesting that part of the initial EB-induced DSBs was repaired (Figure 5A).

The analysis of DSBs by neutral COMET assay is based on the fact that DSBs result in the extension of DNA loops, which form a comet-like tail after neutral electrophoresis of lysed and salt-extracted nuclei [47]. The amount of DNA in the tail of the comets is correlated to the level of DNA damage [48]. While the tail length of the comets derived from LNCaP cells treated for 4 h with EB were comparable to the control, they were significantly increased after EB treatment for 72 h and similar to the doxorubicin control (Figure 5B). The tail length of the comets of MDA-MB-231 cells was significantly increased after EB treatment for 4 h when compared to the control (Figure 5B). Yet, they were visibly shorter when MDA-MB-231 cells were treated with EB for longer periods of time (72 h), suggesting a reduction in the amount of DSBs over time and that part of the initial damage was repaired. In summary, EB induced DNA damage by causing DSBs in LNCaP and MDA-MB-231 cells. Furthermore, both cell lines displayed distinct kinetics of EB-induced DNA damage, suggesting cell line-specific responsive mechanisms.

### EB is a topoisomerase II poison

**Figure 6:**
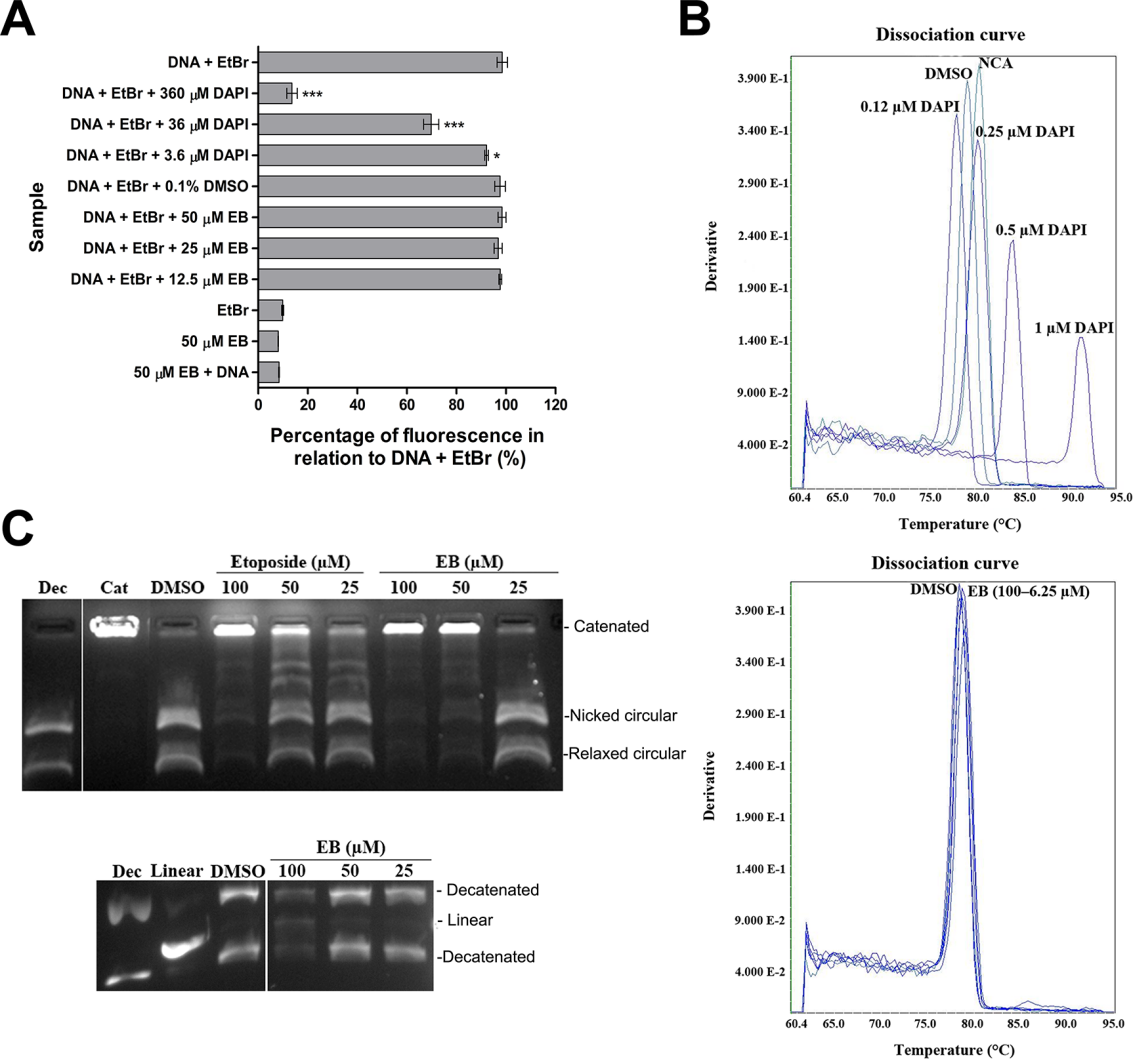
EB inhibited topoisomerase II **(A)** Fluorescent intercalator displacement assay. EB at the indicated concentrations was added to reactions containing plasmid DNA and EtBr, and fluorescence of EtBr was measured (λ_ext_ = 210 nm, λ_em_ = 600 nm) in a FLUOstar Omega plate reader (*n* = 3, mean ± SD). DAPI at the indicated concentrations was used as a positive control for EtBr displacement. Asterisks indicate statistical significant results with p*** < 0.001 and p* < 0.05 in a One-way ANOVA analysis. **(B)** DNA melting temperature analysis. The temperature-dependent dissociation of SYBR^®^ Green-stained double-stranded DNA in the presence of different concentrations of EB (6.25, 12.5, 25, 50 and 100 μM) was monitored on an Applied Biosystems 7900HT Fast Real-Time PCR instrument. DMSO and DAPI (0.12–1 μM) were used as controls. NCA, no compound added. The melting-curves shown are representatives of three replicates. **(C)** Topoisomerase II-mediated decatenation of kDNA in the presence of EB. The indicated concentrations of EB were incubated with topoisomerase II and kDNA, and reaction products were separated and visualized by agarose gel electrophoresis containing EtBr. Etoposide, a topoisomerase II poison, was used as positive control. 0.1% DMSO was used as vehicle control. In the second gel samples were reacted as described above, followed by proteinase K digestion, chloroform/isoamyl alcohol fractionation and agarose gel electrophoresis. The gel was stained with SYBR^®^ Safe. Dec, decatenated kDNA; Linear, linear DNA; Cat, catenated kDNA. For better clarity, irrelevant lanes were removed from the image, as indicated by the gap.

As shown above, EB treatment induced DSBs in LNCaP and MDA-MB-231 cells. In order to verify if the observed DNA damage was a result of a direct interaction of EB with DNA (e.g. DNA intercalation), two different techniques were used. In the first assay, the displacement of ethidium bromide (EtBr) intercalated in double-stranded DNA was measured. The fluorescence emitted by EtBr (excitation at 530 nm and emission at 600 nm) is around 30 times stronger when it is intercalated into DNA. Displacement by a competitor compound will therefore reduce the fluorescence intensity [49, 50]. The second assay measured changes to the melting temperature of double-stranded DNA. In both assays the fluorescent, DNA intercalating compound DAPI was used as a positive control. As shown in Figure 6A, DAPI displaced EtBr from the EtBr-DNA complex in a concentration-dependent manner, as indicated by the strong reduction in fluorescence (Figure 6A). In contrast, EB did not affect the fluorescence of the EtBr-DNA complex even at the highest concentration tested (50 μM), which was almost 100-fold more than EtBr, suggesting that EB did not intercalate in DNA. Next, the thermal profile of double-stranded DNA complexed with fluorescent SYBR^®^ Green was analyzed (Figure 6B). Melting curve analysis comprises the assessment of the dissociation characteristics of double-stranded DNA during heating. The melting point is the temperature at which 50% of the DNA is denatured and present as single-stranded DNA. The interaction of compounds with DNA can stabilize or destabilize its structure, affecting the melting temperature [51–53]. For example, DMSO is known to inhibit secondary structures of DNA and reduces the melting temperature of G/C-rich DNA sequences [54]. Consistent with this, DMSO shifted the melting temperature from 80.39°C to 78.34°C (Figure 6B). The dissociation of double-stranded DNA can be monitored using a DNA-intercalating fluorophore such as SYBR^®^ Green, which fluoresces 1000-fold more intensely when intercalated [55]. Heat-induced denaturation of DNA leads to reduced binding of the fluorophore and can be measured by a reduction in fluorescence [56]. As shown in Figure 6B, the positive control DAPI increased the DNA melting temperature by up to 10°C in a concentration-dependent manner (0.12 – 1.0 μM), indicating a physical interaction of DAPI with DNA which probably stabilized the double helix [57]. Consistent with this, DAPI displaced SYBR^®^ Green from the DNA as indicated by a concentration-dependent reduction of the median fluorescent intensity of the DNA-SYBR^®^ Green complex by up to 2.8-fold. In contrast, EB did not affect the DNA melting temperature or caused displacement of SYBR^®^ Green at all concentrations tested (6.25 – 100 μM, Figure 6B, Table S2). Taken together, both the displacement assay and DNA melting temperature analysis demonstrated that EB did not directly interact with DNA, suggesting that EB induced DNA double strand breaks were possibly mediated indirectly by targeting a DNA modifying enzyme.

Ascidians are a known source for compounds that induce DSBs via inhibition of topoisomerase II (topo II) [58]. TOPO II is an important enzyme that participates in DNA replication, transcription and chromosome condensation. It has the essential role of regulating the uncoiling of DNA through catalyzing transient breaks in the DNA duplex in an ATP-dependent reaction to release topological stress. An *in vitro* assay for TOPO II activity was carried out in order to determine whether EB caused DNA damage by inhibiting TOPO II [59]. EB was incubated with kinetoplast DNA (kDNA) and TOPO II, and the decatenation activity of TOPO II was analyzed by agarose gel electrophoresis (Figure 6C). EB strongly decreased the decatenation of kDNA in a concentration-dependent manner, as judged by the decreased levels of decatenated DNA and increased levels of high molecular weight kDNA with low electrophoretic mobility (Figure 6C). Interestingly, 50 μM of EB were visibly more potent in inhibiting TOPO II than the equimolar concentration of the known TOPO II poison etoposide (Figure 6C). Next, the experiment was repeated in a modified format to test for the presence of linear DNA (covalently bound to TOPO II) which is a product of the cleavage reaction and indicative that EB acted as a TOPO II poison rather than a catalytic inhibitor. As shown in Figure 6C, this analysis demonstrated that, with increasing inhibition of the decatenation reaction, EB generated linearized DNA in a concentration-dependent manner (Figure 6C), indicating that EB is a TOPO II poison.

## DISCUSSION

We previously showed that EB displayed cytotoxicity against MDA-MB-231 cells through induction of apoptosis [3]. Here, we demonstrated that EB significantly arrested MDA-MB-231 and LNCaP cells in the G2 phase after 24 h in a time- and concentration-dependent manner. G2 arrest is regulated by the DNA damage check point, which allows DNA repair by the different repair systems before entering into mitosis [60, 61]. The microarray results with LNCaP cells showed that EB activated pathways related to DNA damage and to G2/M check point regulation. EB-induced DNA damage was confirmed by observations of increased levels of γH2AX foci and a positive COMET assay after 4 h treatment in LNCaP and MDA-MB-231. DSBs can have dangerous consequence for genomic stability and cell survival when not repaired. They may be partly recognized because of the destabilization of chromatin structure, which activates homologous recombination repair (HRR) or non-homologous end joining (NHEJ) as part of the DNA damage response [62–64]. The reduction in the number of DSBs in the breast cancer cell line after a longer incubation period suggests that the DNA was repaired. Nevertheless, this seemed not to have happened in the LNCaP prostate cancer cell line. Gene expression profiling indicated that *BRCA1* and *BRCA2*, which are major players in the repair of DNA DSBs by homologous recombination, were down-regulated by 17- and 12-fold [65]. Genes involved in DSBs repair by NHEJ, such as *PARP1*, *XRCC5*, *PRKDC*, *XRCC1* and *DCLRE1C* were also down-regulated [66]. These are the main DSBs repair systems in the cell, and their inactivation could be the reason for the accumulation of DSBs over time in EB-treated LNCaP cells. In many cases, chemoresistance of cancer cells to DNA damaging agents is because of increased DNA repair. Thus, co-targeting DNA repair mechanisms could cause hypersensitivity to DNA damage [67–69]. With this aim different compounds that target components of the DNA repair machine have been developed, such as O6-alkylating agents and temozolomide [70].

DNA damage is first detected by ATM, ATR and DNA-PK and can induce cell cycle arrest to allow DNA repair, or induce senescence or apoptosis. The arrest at G2/M phase prevents mitotic segregation of damaged chromatids and is mediated by ATM/ATR, CHK1 and p21^CIP1/WAF1^ [71–73]. Induction of the CDK inhibitor p21^CIP1/WAF1^ is required for nuclear sequestration of inactive cyclin B-Cdc2 complexes, leading to cell cycle arrest at G2. CHK1 activation through phosphorylation at Ser345 and Ser317 is induced by ATR, after phosphorylation at Ser286 and Ser301 by CDKs for an efficient response to DNA damage [74]. Active CHK1 inactivates CDC25C by phosphorylation, impairing cell progression to mitosis, since it is responsible to activate CDC2 by removing the inhibitory phosphate groups Thr14/Tyr15 [75]. All cancer cells have a defect in G1 control and this makes them extremely dependent on S and G2/M checkpoints [76, 77]. Our microarray data showed that *CHEK1* (CHK1) was down-regulated by 13-fold and *CDKN1A* (p21^CIP1/WAF1^) was up-regulated by 12-fold in LNCaP cells treated with EB. The increase of *CDKN1A* was confirmed by qRT-PCR for LNCaP and MDA-MB-231 cells. Western blot results displayed that CHK1 was activated through phosphorylation at Ser345 after 24 h treatment of MDA-MB-231 cells with EB. The maximum amount of p-CHK1 was observed after 48 h treatment. Nevertheless, it has been reported that CHK1 is dispensable in the presence of a functional p21^CIP1/WAF1^ induction [77].

Transcription of *TP53* and phosphorylation or stabilization of p53 protein was not observed in treated LNCaP cells but Ingenuity pathway analysis predicted *TP53* as an activated upstream regulator with a z-score of 9.74 (data not shown). Moreover, *TP53I3*, which is directly regulated by *TP53* was 48-fold up-regulated after treatment. In contrast, EB-treated MDA-MB-231 cells increased phosphorylation and total p53 protein in a time dependent way. Phosphorylation of p53 at Ser15 is mediated by ATM and CHK2 in response to DNA damage [78]. Phosphorylation also occurs at Ser20 or Ser37 and promotes the stabilization and activation of p53. Protein p53 is one of the inducers of the expression of p21^CIP1/WAF1^ and GADD45A, which were both up-regulated in LNCaP cells treated with EB as shown by microarray. Nevertheless, the data presented here suggested that the induction of p21^CIP1/WAF1^ and GADD45A in LNCaP cells was p53-independent. Cell cycle arrest induced by p21^CIP1/WAF1^ has been previously described by both p53-dependent and independent pathways [79–81]. Apart from the tumor suppressor p53, p21^CIP1/WAF1^ can also be regulated by BRCA1 [82], CHK2 [83], and others.

Despite the 5-fold down-regulation of *CHEK2* observed by microarray in LNCaP cells, an increased activation of CHK2 by phosphorylation at Thr68 was noticed. The same was observed in EB-treated MDA-MB-231 cells. This activation is mediated by ATM and induces CHK2 dimerization [84]. After intermolecular phosphorylation, enzymatically active monomers leave chromatin to phosphorylate different substrates; including CDC25C that together with CHK1 leads to cell cycle arrest at G2/M phase [85, 86]. CHK2's role in G2/M arrest is not well defined. It is possible that CHK2 activation is redundant in the presence of other checkpoint regulators [87]. CHK2 function could also be associated in controlling other proteins involved in the cell cycle, such as phosphorylating RB [88]. The CHK2 kinases inactivate CDC25 via phosphorylation at Ser216, blocking the activation of CDC2. The complex CDC2/CYCLIN B is of fundamental importance to the progress from G2 into mitosis. CDC2 is kept inactive during G2 phase through phosphorylation at Thr14/15 by WEE1 and MYT1 protein kinases [89–93]. The down-regulation of *CDK1* (CDC2) gene expression (19-fold) in LNCaP cells was confirmed on the protein level by Western blot. After 24 h treatment the expression levels of CDC2 decreased dramatically, followed by loss of p-CDC2. In contrast, CDC2 protein accumulated in EB-treated MDA-MB-231 cells. However, this was accompanied by a greater increase in inhibitory CDC2 phosphorylation, suggesting that CDC2 activity overall was suppressed. Microarray and qRT-PCR showed that the expression of *CCNB1* (CYCLIN B) was down-regulated in MDA-MB-231 and LNCaP cells. Thus, the G2/M arrest after EB treatment of MDA-MB-231 cells was induced ultimately by inactivation of cdc2 and down-regulation of CYCLIN B, along with CHK1 activation and p21 expression induced by p53 stabilization and activation. Another contribution for the G2/M arrest in LNCaP cells might have been *GADD45A* and *GADD45G* which were up-regulated after EB treatment and have been shown to inactivate CDC2/CYCLIN B kinase [94]. Thus, the results indicated that EB induced G2 arrest in LNCaP cells by down-regulation of CDC2 and CYCLIN B expression, which was maintained through up-regulation of GADD45 and p21^CIP1/WAF1^.

Studies have shown that overexpression of p21^CIP1/WAF1^ is related to induction of BAX and promotion of apoptosis [95, 96]. Consistent with this, EB induced apoptosis in the breast cancer cell line. Cell cycle distribution of treated MDA-MB-231 cells revealed an increase in the sub-G1 population, demonstrating that EB induced cell death. EB-induced apoptosis in MDA-MB-231 cells was confirmed by the detection of PARP cleavage. Nevertheless, high levels of p21^CIP1/WAF1^ expression can also inhibit apoptosis through inhibition of PROCASPASE 3 activity [97], stabilization of the anti-apoptotic protein c-IAP1 [98], or down-regulation of caspase-2 [99]. These anti-apoptotic effects of p21^CIP1/WAF1^ might explain why EB did not induce cell death in LNCaP cells when treated for up to 10 days.

DSBs may be caused directly (replication/transcription-independent) or indirectly (replication/transcription-dependent) by cytotoxic compounds [68]. SSBs can become DSBs when a replication fork meets a SSB [100]. Similarly, collisions of RNA polymerase during transcription with TOPO II/DNA complexes can cause DSBs [101]. The induction of DSBs and activation of the DNA damage pathways by EB could have been due to a direct interaction of EB with DNA, such as binding or intercalation, induction of oxidative stress response or inhibition/poison of topoisomerases. EtBr displacement assay and DNA melting temperature analysis strongly suggested that EB did not directly interact with DNA. Instead, EB was found to inhibit TOPO II activity *in vitro* and to stabilize the cleavage complex. Microarray analysis showed that the expression of *TOP2A* was down-regulated by 49-fold, whereas transcription of the isoform *TOP2B* was only reduced by 1.3-fold. While *TOP2A* is cell cycle regulated by Rb and important for DNA synthesis and chromosome segregation; [102, 103]. *TOP2B* is mainly involved in transcription and has been shown to bind to the androgen receptor [104]. Thus, our findings indicate that EB is a topoisomerase II poison that, like etoposide, does not directly interact with DNA [105, 106].

It has been shown that BRCA1 is necessary for ubiquitination of topoisomerase IIα, which is correlated with higher DNA decatenation activity. Decatenation of chromatid arms happens before mitosis, while centromeric catenations persist till metaphase/anaphase [107, 108]. Any problem during this process activates the decatenation G2 checkpoint signaling and can lead to G2 arrest in the absence of DNA damage [109, 110]. Our results indicate down-regulation of *BRCA1*, which could result in defective DNA decatenation [111]. Hence, DSBs and inhibition of chromatid decatenation caused by topo II poisoning might have caused the G2 arrest in EB-treated cells.

Besides its cytotoxicity towards LNCaP and MDA-MB-231, EB showed to be cytotoxic to the non-malignant cell lines RWPE-1 and NFF. It is known that rapidly proliferating cells, such as RWPE-1 and NFF, are more sensitive to TOPO II inhibitors because they contain high concentrations of topoisomerase II, especially the α isoform [112–114]. Nevertheless, it has been reported that intrinsic characteristics of the cell line can also affect sensitivity to TOPO II catalytic inhibitors. For example, researchers have found that BRCA1 mutant cells are more sensitive to TOPO II catalytic inhibitors [18]. Moreover, defects in the G2/M checkpoint that regulates cell cycle by controlling the presence of catalytic TOPO II can also affect cell sensitivity [115–117].

Natural products are still the main source of topoisomerase II-targeting agents, and they usually contain polycyclic, aromatic, or planar structures and intercalate DNA [28]. EB was shown to be a non-intercalating topoisomerase II poison that arrests LNCaP and MDA-MB-231 cells at the G2 phase. Similar results were obtained with the treatment of MDA-MB-231 cells with the topoisomerase IIα inhibitor CS1. CS1 was less toxic than etoposide and showed potential anti-multidrug resistance capabilities [118]. Further tests will determine EB toxicity and its preference for topoisomerase IIα or β isoform. Different strategies have been used to increase the potency and selectivity of topoisomerase II-targeting drugs. The development of compounds more specific to the α isoform can reduce adverse effects such as, cardiotoxicity and secondary malignancies. Another approach is the use of different drug delivery systems (e.g. polyethylene glycol and nanoparticles) to target tumors while sparing normal tissues or increase drug activity [119]. In order to increase the potency, drug combination approaches have revealed positive results. The use of PARP inhibitors are likely to be beneficial in specific tumors, such as in BRCA1-positive breast cancer cells [120]. Finally, combination treatment of doxorubicin with microRNA-21 inhibitor resulted in increased expression of tumor suppressor genes, increasing synergistically the anti-cancer activity of doxorubicin towards glioma *in vitro* [121].

In summary, our work shows that the natural product eusynstyelamide B (EB) is a novel topoisomerase II poison with comparable potency to the anti-cancer drug etoposide. Our findings warrant further studies investigating the efficacy of EB in various cancer models and potential synergies with clinically used anti-cancer drugs.

## MATERIALS AND METHODS

### Reagents

A stock solution of 10 mM EB was prepared in DMSO (Sigma-Aldrich) and sonicated to provide complete dissolution of the natural product. For cell treatment, the stock solution was diluted to the desired concentration in the appropriate complete cell culture medium. Tunicamycin, etoposide, chloroquine, taxol, and nocodazole were purchased from Sigma-Aldrich and dissolved in DMSO. Doxorubicin (Sigma-Aldrich) was dissolved in water.

### Antibodies

The antibodies used in this study are described in Supplementary Table S3.

### Cell culture

LNCaP and MDA-MB-231 cells were obtained from the American Type Cell Culture Collection. LNCaP cells were maintained in phenol-red free RPMI-1640 medium (Life Technologies) supplemented with 5% fetal calf serum (FCS) (Life Technologies) at 37°C in an atmosphere containing 5% CO_2_. MDA-MB-231 cells were cultured in DMEM supplemented with 10% (v/v) FCS (Life Technologies).

### Live cell analysis with xCELLigence and IncuCyte technologies

For real-time measurement of the cell index, which is a composite figure of cell number, morphology and adhesiveness, and computation of IC_50_, cells were analyzed on a xCELLigence system (Roche) as described previously.[122] LNCaP (1.0 × 10^4^ cells per well), NFF (1.8 × 10^3^ cells per well) and RWPE-1 cells (4.0 × 10^3^ cells per well) cells were seeded in triplicate in 96-well E-plates^®^ for 24 h. Cells were treated with the indicated concentrations of EB for 72 h, and the cell index measured hourly for 96 h. Calculations of IC_50_ (72 h) from three independent experiments were performed with GraphPad Prism (GraphPad Software). For real-time live cell imaging, LNCaP cells were seeded in 96-well plates at 4.0 × 10^3^ cells per well and grown to 20% confluence before addition of the indicated concentrations of EB or tunicamycin (1 μg/mL). Growth as a function of increasing confluence was monitored in real-time by phase contrast microscopy with the IncuCyte FLR system (Essen BioScience). Images were captured with a 10 × objective at 2 h intervals from 3 separate wells per treatment for 96 h, and mean ± SD of confluence percentages was computed.

### Cell cycle analysis

LNCaP (2.0 × 10^5^ cells per well) and MDA-MB-231 (1.0 × 10^5^ cells per well) cells were seeded in a 6-well plate for 24 h. For time course studies, cells were treated with 2.5 μM EB (MDA-MB-231) or 5.0 μM EB (LNCaP) for the indicated times. For the 10 day treatment with EB, LNCaP cells (2.5 × 10^4^) were treated with 5 μM EB for 72 h followed by periodic change of growth medium. For dose titration studies, LNCaP and MDA-MB-231cells were treated with the indicated concentrations of EB for 72 h. Cells were processed, and DNA content was analyzed by flow cytometry as described elsewhere [123]. The percentage of cells in each cell cycle phase was calculated with ModFit LT (Verity Software House) based on DNA histograms of 20,000 cells per treatment.

To calculate the mitotic index, LNCaP (1.0 × 10^4^ cells per well) and MDA-MB-231 (5.0 × 10^3^ cells per well) cells were seeded in a 96-well plate for 24 h. Cells were treated with 5 μM EB, 0.1% DMSO or 83 nM nocodazole for 24 h. The cell culture medium was removed, and cells were fixed in 4% paraformaldehyde followed by incubation with blocking buffer (2% BSA in PBS). Cells were then reacted with anti-phospho-histone H3 (Ser10, 1:100, Abcam) overnight, and DNA counter stained with DAPI (1:500, Life Technologies) and Alexa Fluor^®^ 568 donkey anti-rabbit IgG (1:500, Life Technologies). Samples were analyzed using the high-content screening machine Operetta (PerkinElmer). The mitotic index was calculated with Harmony^®^ software (PerkinElmer). Statistical significance (*n* = 3, mean ± SD) was analyzed with GraphPad Prism (GraphPad Software) by one-way ANOVA with Dunnett's multiple comparison test.

### Microarray gene expression profiling

For sample preparation, LNCaP cells were seeded at a density of 2.0 × 10^5^ cells per well in a 6-well plate. After 24 h, cells were treated with 5 μM EB or 0.1% DMSO for 24 h. Triplicates of each condition were prepared for microarray profiling as previously described [124].

The microarray raw data were processed using the Agilent Feature Extraction Software (v10.7) as described elsewhere [124]. Genes that were significantly different between two groups were identified with an adjusted *p*-value of ≤ 0.05, and an average fold change of ≥ 1.5. The gene expression data have been submitted to Gene Expression Omnibus (GEO) with the accession number GSE74212. The filtered gene lists were examined by Ingenuity Pathway Analysis (IPA, Ingenuity Systems Inc.) for functional annotation and gene network analysis.

### Quantitative real time polymerase chain reaction (qRT-PCR)

LNCaP (2.0 × 10^5^) and MDA-MB-231 (1.0 × 10^5^) cells were seeded in a 6-well plate for 24 h and treated with 5 μM EB or 0.1% DMSO for 24 h. Total RNA was obtained using the RNeasy mini kit (Qiagen) according to the manufacturer's instructions. The quantity and the quality of the RNA were measured using a Nano-drop UV spectrophotometer (ThermoFisher Scientific). The samples were treated with DNAse I (Life Technologies), and cDNA was prepared from 2.0 μg total RNA with Superscript III (Life Technologies). Quantitative real-time PCR (qRT-PCR) was performed with SYBR Green PCR Master Mix (Life Technologies) on a 7900HT Fast PCR System (Applied Biosystems). Data were analyzed with SDS2.3 software (Applied Biosystems). mRNA expression levels were calculated by the ΔΔCt method and normalized relative to the expression levels of the house keeping gene (*RPL32*) of the respective treatment and calculated relative to the vehicle control (DMSO). Statistical significance (*n* = 3, mean ± SD) was analyzed with GraphPad Prism (GraphPad Software) by Student's *t* test. The sequences of the primers used are listed in the Supporting Information (Table S4).

### Western blotting

LNCaP (1.5 × 10^5^ cells per well) and MDA-MB-231 (5.0 × 10^4^ cells) were seeded in a 6-well plate and treated for the indicated times with 5.0 and 2.5 μM EB, respectively. As positive controls, cells were treated with doxorubicin (1 μM, 48 h), etoposide (25 μM, 24 h), chloroquine (25 μM, 48 h), taxol (2 nM, 24 h), or nocodazole (83 nM, 24 h). 0.1% DMSO was used as vehicle control. At the end of the treatment, cells were harvested and lyzed with lysis buffer containing protease inhibitor cocktail (Roche) and phosphatase inhibitors [124, 125]. Protein concentration was determined through a bicinchoninic protein assay (BCA assay, Thermo Fisher Scientific). Thirty micrograms of protein lysates per well were loaded onto a NuPAGE^®^ 4–12% Bis-Tris Gel (Life Technologies) and transferred to a nitrocellulose membrane by wet transfer. After primary antibodies and secondary HRP conjugated secondary antibody incubation (Table S4), membranes were developed with a chemiluminescent HRP Substrate (Immobilon Merck Millipore). Beta-ACTIN was used as a loading control. Protein signals were quantified using Image Lab^™^ software (Bio-Rad), normalized to the respective loading control, and expressed relative to the control treatment. Phosphorylation levels were calculated relative to the normalized total amount of the respective protein.

### DNA damage analysis

For quantitative analysis of γH2AX foci formation by fluorescence microscopy, LNCaP (6.0 × 10^4^ cells per well) and MDA-MB-231 cells (3.0 × 10^4^ cells per well) were seeded on glass coverslips (coated with poly-l-ornithine for LNCaP) in a CO_2_ humidified incubator for 24 h. Cells were treated with 0.1% DMSO, 5.0 μM EB, or 1.0 μM doxorubicin for the indicated times. Cells were fixed with 4% paraformaldehyde (Sigma-Aldrich) and stained as described previously with some minor modifications.[126] Cells were probed with anti-phospho-histone H2AX (Ser139) antibody (1:500, Merck KGaA) at 4°C overnight, followed by Alexa Fluor^®^ 488 goat anti-mouse IgG (1:500, Life Technologies) and DNA counterstaining with DAPI (1:500, Life Technologies). γH2AX foci were imaged with a DeltaVision microscope (GE Healthcare) and counted with MetaMorth software (Molecular Devices). A minimum of 100 cells were analyzed per sample.

For the analysis of DNA damage by neutral comet assay, LNCaP (2.0 × 10^5^ cells per well) and MDA-MB-231 (1.0 × 10^5^ cells per well) were seeded in a 6-well plate for 24 h. Cells were treated with 5.0 μM EB, 1.0 μM doxorubicin or 0.1% DMSO for the indicated times. The assay was performed as described previously with minor modifications [127]. Briefly, cells were washed once with DPBS (Invitrogen) and harvested to obtain a single cell suspension of 1.0 × 10^6^ cells/mL in 1x Tris-borate-EDTA (TBE, 89 mM Tris Base, 89 mM Boric acid, 2 mM EDTA). Approximately 4.0 × 10^3^ cells were suspended into 150 μl of 0.6% low melting-point agarose (Sigma-Aldrich) and 60 μl of this mixture was applied onto each well of a COMET slide (Trivigen Inc). Slides were then placed on a cold surface (4°C) for 10 min followed by immersion in ice cold lysis buffer (2.5 M NaCl, 100 mM EDTA, 10 mM Tris, 1% Triton-X-100; pH 10) overnight. Slides were washed in 100 mL of 1 × TBE for 15 min at 4°C. After removing excess salts with 1 × TBE, slides were placed in an electrophoresis tank (BioRad) containing 1 × TBE buffer. Electrophoresis was performed for 30 min at 70 V and 90 mA by adjusting the height of the buffer. After the completion of electrophoresis, slides were immersed in distilled water for 5 min followed by 5 min incubation in 70% ethanol. Slides were then incubated at 45°C for about 10 min and left to cool down at RT. 100 μl of DAPI (1 μg/mL) we added onto each well and incubated at 4°C for 5 min. Excess DAPI was removed and slides were scanned using an epifluorescence microscope (Nikon Eclipse) equipped with a 10 × objective. One hundred comets in each sample were scored using the COMET Score software (TriTek Corp). Statistical significance (mean ± SD) was analyzed with GraphPad Prism (GraphPad Software) by one-way ANOVA with Dunnett's multiple comparison test.

### DNA interaction studies

An EtBr displacement assay was performed to identify the ability of EB to intercalate with DNA. As a positive control, increasing concentrations of the DNA intercalator DAPI (0.04–360 μM) were tested. Increasing concentrations of EB (6.25–50 μM) were incubated with 2.5 μg plasmid DNA in the presence of 1.3 μM EtBr in a 96-well plate completed with TE buffer (10 mM Tris-Cl, pH 7.5 and 1 mM EDTA) to a final volume of 100 μl. Controls consisted of the individual reaction components (EtBr, DNA, and EB) and EtBr mixed with DNA. Each sample was set up in triplicate, and fluorescence was measured in a FLUOstar Omega plate reader (BMG Labtech) with an excitation at 530 nm and emission recorded at 605 nm. Readings were corrected for background fluorescence.

To study DNA binding of EB, increasing concentrations of EB (6.25 – 100 μM), DMSO (0.1%), or DAPI (0.12–1.00 μM) were added in triplicate to a completed qRT-PCR reaction run with SYBR^®^ Green PCR Master Mix (Life Technologies) containing a 151 bp PCR product of the RPL32 gene. Melting curves were generated with an Abi 7900HT qRT-PCR machine (Applied Biosystems) using the protocol: 50°C 2 min, 95°C 15 s, 60°C 15 s, and 95°C 15 s with data recording of the temperature gradient between 60°C and 95°C. Thermal profiles were analyzed with SDS 2.4 software (Applied Biosystems).

### Topoisomerase II assay

The topoisomerase II-mediated decatenation of kinetoplast DNA in the presence of EB (25-100 μM), etoposide (25-100 μM), or DMSO as vehicle control was carried out using the kDNA based Topoisomerase II Drug Screening kit (TopoGen) as described by the manufacturer. Samples were separated on a 1% (w/v) agarose gel by electrophoresis for 30 min at 100 V and visualized by EtBr staining under UV light with a Quantum ST4 (Vilber Lourmat) gel documentation system. To detect the linear DNA intermediate of the topoisomerase II reaction, samples were prepared and reacted as described above followed by proteinase K treatment (50 μg/mL, Sigma-Aldrich) for 15 min at 37°C. DNA was extracted by chloroform/isoamyl alcohol extraction (24:1, Sigma-Aldrich) before electrophoresis on a 1% (w/v) agarose gel containing SYBR^®^ Safe (Life Technologies).

### Appendix A. supplementary material

The following are supplementary material related to this article.

## SUPPLEMENTARY MATERIAL FIGURES AND TABLES


