# Supplementary material for: The ascidian natural product eusynstyelamide B is a novel topoisomerase II poison that induces DNA damage and growth arrest in prostate and breast cancer cells
Source: Oncotarget. 2015 Nov 2;6(41):43944–63. doi: 10.18632/oncotarget.6267 (PMC4791278; doi:10.18632/oncotarget.6267)
Supplement: Supplementary file 1 [file oncotarget-06-43944-s001.pdf]

The ascidian natural product eusynstyelamide B is a novel topoisomerase II poison that induces DNA damage and growth arrest in prostate and breast cancer cells

Supplementary Materials

Cell Culture

NFF and RWPE-1 cells were kind gifts from D. Zencak and G.P. Risbridger, respectively. NFF cells were cultured in DMEM supplemented with 10% (v/v) FCS (Life Technologies). RWPE-1 cells were grown in Keratinocyte-SMF medium supplemented with human Epidermal Growth Factor (EGF 1–53) and bovine Pituitary Extract (BPE) (Life Technologies).

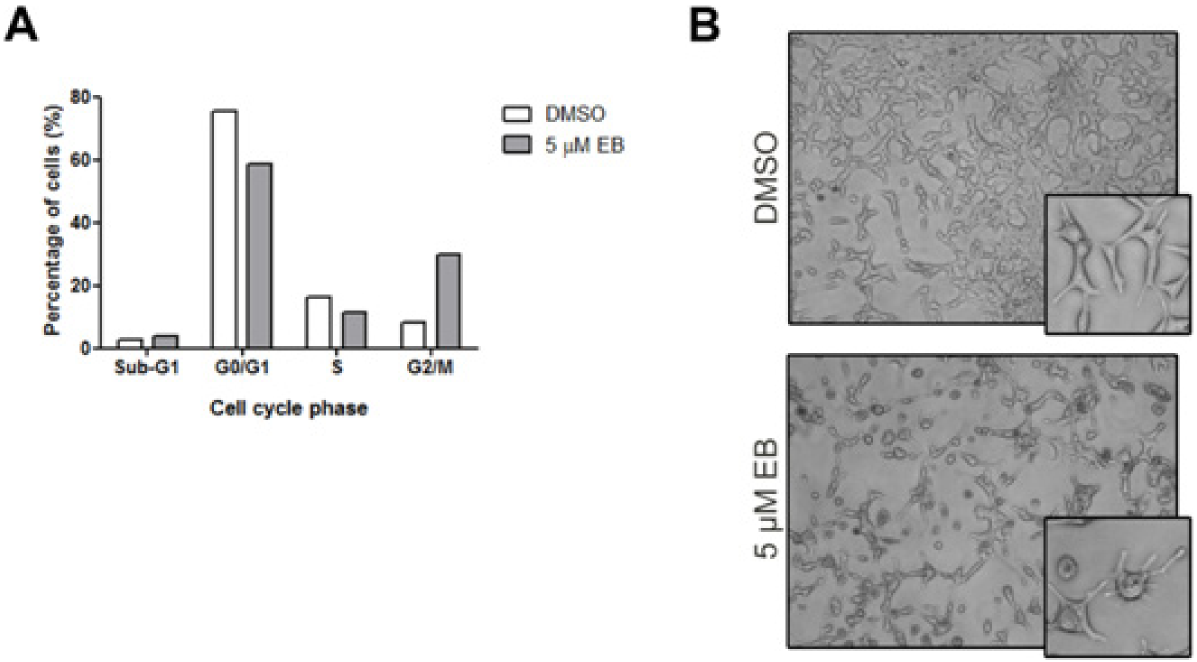

Supplementary Figure S1: LNCaP cells did not die after 10 days of EB treatment. (A) Cell cycle distribution of LNCaP cells after 10 days treatment with 5 µM EB or 0.1% DMSO (control). Cells were stained with PI, and DNA content analyzed by flow cytometry using a FACSCanto instrument (BD Biosciences). **(B)** Cell morphology of LNCaP cells after 10 days treatment with 5 µM EB or 0.1% DMSO (control).

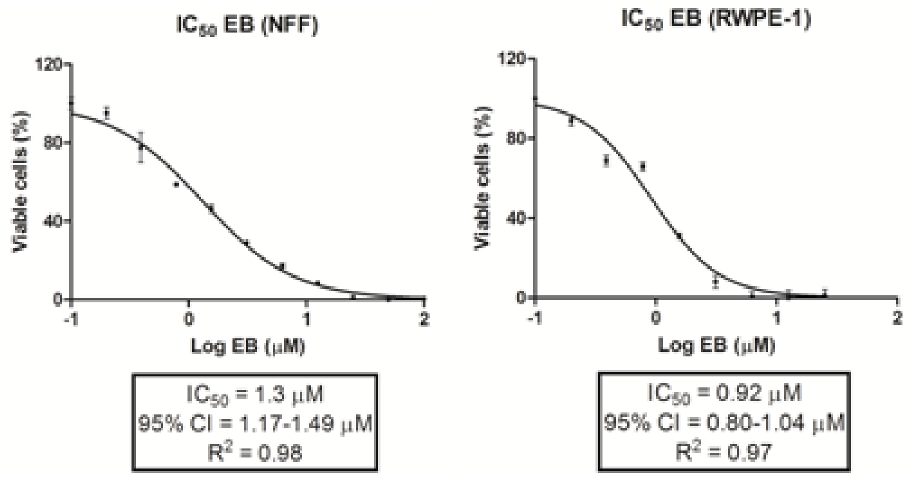

Supplementary Figure S2: Determination of the IC_{50} of EB in (A) NFF cells and in (B). non-malignant RWPE1 prostate cells. Both cell lines were seeded for 24 h and treated with different concentrations of EB for 72 h. Cell growth was monitored using RTCA (xCELLigence System, Roche), and the CI was used to calculate the IC_{50} along with 95% confidence interval (CI) and R^2 with GraphPad Prism 5 software.

Supplemental Table S1: Top 20 differentially regulated genes by EB treatment in LNCaP cells

Gene	Fold change	Biological role
SULF2	↑61.4	cell signalling, expression in proliferation and abnormal cell morphology, activation in apoptosis
TP53I3	↑47.8	induction of apoptosis by oxidative stress
IGF2	↑23.5	proliferation, differentiation, growth, apoptosis, survival
CYP4B1	↑21.7	drug metabolism and synthesis of cholesterol, steroids and other lipids
APOBEC3H	↑17.7	salvage pathways of pyrimidine ribonucleotides
TGFB1	↑13.4	cell proliferation and inhibition of cellular adhesion
KANK3	↑13.2	negative regulation of Stress fiber assembly
CDKN1A	↑12.4	cell cycle regulation
SERPINB5	↑11.6	participates in apoptosis, motility, adhesion invasion, and proliferation
APOD	↑11.0	response to reactive oxygen species
NCAPG	↓58.0	condensation and stabilization of chromosomes during mitosis and meiosis
HMMR	↓56.9	metabolism and cytoskeleton organization
CDC20	↓56.9	required for two microtubule-dependent processes, nuclear movement prior to anaphase and chromosome separation
PBK	↓53.4	protein phosphorylation and mitosis
NDC80	↓51.1	organize and stabilize microtubule-kinetochore interactions and is required for proper chromosome segregation
HJURP	↓51.0	CenH3-containing nucleosome assembly at centromere; chromosome segregation
TOP2A	↓49.3	chromosome condensation; chromosome segregation; DNA replication and repair
UBE2C	↓49.1	activation of anaphase-promoting complex activity and mitotic cell cycle spindle assembly checkpoint
SPC25	↓48.8	kinetochore-microtubule interaction and spindle checkpoint activity
BIRC5	↓48.7	G2/M transition of mitotic cell cycle

Up-regulated genes are indicated by ↑ and down-regulated genes are indicated by ↓

Supplemental Table S2: List of DNA melting temperatures in the presence of the indicated compounds. NCA, no compound added (control)

Sample	Tm (°C)	ΔT
NCA	80.35 ± 0.15	0.00
0.1 %DMSO	78.80 ± 0.28	−1.55
1 µM DAPI	91.45 ± 0.25	11.10
0.5 µM DAPI	84.20 ± 0.10	3.85
0.25 µM DAPI	80.40 ± 0.10	0.05
0.12 µM DAPI	78.35 ± 0.25	−2.00
100 µM EB	78.67 ± 0.21	−0.13
50 µM EB	78.50 ± 0.29	−0.30
25 µM EB	78.67 ± 0.21	−0.13
12.5 µM EB	78.30 ± 0.24	−0.50
6.25 µM EB	79.07 ± 0.09	0.27

The melting temperatures are shown as the mean ± SD (n = 3)

Supplemental Table S3: Details of the primary antibodies used to investigate the mechanism of action of EB

Antibody	Dilution	Isotype	Brand
Phospho-CDC2 (Tyr15)	1:1000	Rabbit IgG	Cell Signaling Technology
Phospho-CHK1 (Ser345)	1:1000	Rabbit IgG	Cell Signaling Technology
Phospho-CHK2 (Thr68)	1:1000	Rabbit IgG	Cell Signaling Technology
Phospho-RB (Ser795)	1:1000	Rabbit IgG	Cell Signaling Technology
Phospho-RB (Ser807/811)	1:1000	Rabbit IgG	Cell Signaling Technology
Phospho-p53 (Ser15)	1:1000	Rabbit IgG	Cell Signaling Technology
Anti-rabbit IgG, HRP-linked	1:5000	Goat	Merck Millipore
Phospho-histone H3 (Ser10)	1:1000	Rabbit IgG	Abcam
CDC2 (POH1)	1:1000	Mouse IgG2a	Cell Signaling Technology
PARP (46D11)	1:1000	Rabbit IgG	Cell Signaling Technology
CDC25A (144)	1:1000	Rabbit IgG	Santa Cruz Biotechnology
LC3B	1:1000	Rabbit IgG	Cell Signaling Technology
Beta-ACTIN (13E5)	1:3000	Rabbit IgG	Cell Signaling Technology
Anti-mouse IgG, HRP-linked	1:5000	Sheep IgG	Cell Signaling Technology
Alexa Fluor® 568 anti-rabbit IgG	1:500	Donkey	Life Technologies

Supplemental Table S4: Sequences of the sense and antisense primers and the concentrations used for qRT-PCR

Genes	Primer pairs	Concentration (µM)
CDK1	5′-GTCAGCTCGTTACTCAACTCC<br>5′-CCACACTTCATTATTGGGAGTG	0.2<br>0.2
CCNB1	5′-AGAGCCATCCTAATTGACTG<br>5′-CAACCAGCTGCAGCATCTTC	0.2<br>0.2
CDKN1A	5′-GTGGACCTGTCACTGTCTTG<br>5′-CCTCTTGGAGAAGATCAGCC	0.2<br>0.2
CDC25A	5′-TGAATGGCAAGTTTGCCAACC<br>5′-GTAGGTACAATGGGCTTCTTC	0.2<br>0.2
MKI67	5′-CAAATTACAAGACTCGGTCCCTG<br>5′-GGGAGGTCTTCATGGGCTTC	0.2<br>0.2
GADD45A	5′-CTGGAGAGCAGAAGACCGAAAG<br>5′-GCAGGCACAACACCACGTTA	0.4<br>0.4
GADD45G	5′-GAGTCAGCCAAAGTCTTGAACGT<br>5′-CGCAGCCAGCACACAGAA	0.4<br>0.4
